# Supplementary material for: Novel Angiotensin-Converting Enzyme-Inhibitory Peptides Obtained from Trichiurus lepturus: Preparation, Identification and Potential Antihypertensive Mechanism
Source: Biomolecules. 2024 May 15;14(5):581. doi: 10.3390/biom14050581 (PMC11117660; doi:10.3390/biom14050581)
Supplement: Supplementary file 1 [file biomolecules-14-00581-s001.zip › biomolecules-2956373-supplementary.pdf]

Supplemental Information

Novel Angiotensin-Converting Enzyme-Inhibitory Peptides Obtained from *Trichiurus lepturus*: Preparation, Identification and Potential Antihypertensive Mechanism

Jiaming Cao [1,2], Boyuan Xiang [1,2], Baojie Dou [1,2], Jingfei Hu [1,2], Lei Zhang [1,2], Xinxin Kang [1,2], Mingsheng Lyu [1,2] and Shujun Wang [1,2,*]

[1] Jiangsu Key Laboratory of Marine Bioresources and Environment/Jiangsu Key Laboratory of Marine Biotechnology, Jiangsu Ocean University, Lianyungang 222005, China; jmcao@jou.edu.cn (J.C.); xby@jou.edu.cn (B.X.); bjdou@jou.edu.cn (B.D.); jfhu123@jou.edu.cn (J.H.); leizhang@jou.edu.cn (L.Z.); kangxinxin@jou.edu.cn (X.K.); mslyu@jou.edu.cn (M.L.)
[2] Co-Innovation Center of Jiangsu Marine Bio-Industry Technology, Jiangsu Ocean University, Lianyungang 222005, China
* Correspondence: sjwang@jou.edu.cn

Table S1 the characteristis of peptides

Peptide Sequence	SVM score	Prediction	Hydrophobicity	Hydropathicity	Hydrophilicity	Charge	Mol wt
KRIEAPPHIF	1.79	AHT	-0.17	-0.47	0.19	1	1207.56
AEPVPGKM	1.7	AHT	-0.08	-0.39	0.34	0	828.1
IFPRNPP	1.45	AHT	-0.18	-0.79	-0.16	1	840.07
IPGPPTGPIK	1.4	AHT	0.02	-0.28	-0.1	1	976.31
VPIGAFK	1.38	AHT	0.16	1.06	-0.47	1	730.99
VPIGAFK	1.38	AHT	0.16	1.06	-0.47	1	730.99
RIEAPPHIF	1.36	AHT	-0.07	-0.09	-0.12	0	1079.37
EAPPHIF	1.35	AHT	0.06	-0.11	-0.33	-1	810
FDKPVSP	1.31	AHT	-0.15	-0.63	0.33	0	788.9

							8
PGAPGSPGMP	1.28	AHT	0.04	-0.47	-0.15	0	867.12
FDKPVSPLF	1.27	AHT	0.01	0.24	-0.22	0	1049.34
KAEPVPGKM	1.25	AHT	-0.19	-0.78	0.63	1	956.29
FAPPAPNGVP	1.19	AHT	0.09	0.03	-0.48	0	966.23
LPPIAF	1.19	AHT	0.33	1.62	-1.1	0	656.88
NFPPGPPGIP	1.18	AHT	0.07	-0.5	-0.41	0	992.27
IEAPPHIF	1.17	AHT	0.14	0.46	-0.51	-1	923.17
ILPPGPPTPW	1.16	AHT	0.13	-0.17	-0.74	0	1074.42
FDKPVSPL	1.13	AHT	-0.07	-0.07	0.06	0	902.15
KWEAP	1.12	AHT	-0.23	-1.62	0.42	0	629.78
VILPVPAF	0.94	AHT	0.38	2.26	-1.2	0	855.18
GPTGPAGPR	0.8	AHT	-0.16	-1.04	0.23	1	809.01
GRPGPPGVP	0.77	AHT	-0.11	-0.88	0.17	1	833.08
FINPDPI	0.73	AHT	0.08	0.23	-0.41	-1	815.01
PAAKPLGDL	0.73	AHT	-0.03	0.02	0.16	0	881.14
GPAGPAGPR	0.69	AHT	-0.11	-0.77	0.22	1	778.98
FLGLMWF	0.54	AHT	0.34	1.34	-1.54	0	863.1

LP							8
QGPIGPR	0.53	AHT	-0.22	-1.07	0.2	1	723.92
GAQGPIGPR	0.43	AHT	-0.13	-0.68	0.1	1	852.08
KDPIDPPW	0.37	AHT	-0.21	-1.51	0.48	-1	967.19
KAEPTPGKM	0.34	AHT	-0.27	-1.32	0.76	1	958.26
AWKPPLQ	0.29	AHT	-0.11	-0.84	-0.36	1	839.1
NFSLDGPIP	0.26	AHT	0.03	-0.03	-0.29	-1	959.17
AGFAGDDAPR	0.24	AHT	-0.16	-0.57	0.5	-1	976.13
DQLLHPT	0.24	AHT	-0.14	-0.7	-0.19	-1	823
FAGDDAPR	0.23	AHT	-0.25	-0.89	0.69	-1	847.97
GSPRAPEGAP	0.04	AHT	-0.2	-1.08	0.53	0	938.13
GVDNPGHPFI	0.01	AHT	0.03	-0.27	-0.31	-1	1052.29
HLPPPPP	-0.01	Non-AHT	-0.03	-1.06	-0.33	0	753.98
VDNPGHPF	-0.05	Non-AHT	-0.07	-0.85	-0.16	-1	882.05
DPLYPPGPPK	-0.43	Non-AHT	-0.15	-1.33	0.19	0	1080.37
AAPEPAPAPK	-0.68	Non-AHT	-0.1	-0.66	0.4	0	948.2
TEAPLNPK	-0.7	Non-AHT	-0.24	-1.15	0.44	0	869.08
IAGPKELGL	-0.83	Non-AHT	0.06	0.46	0.01	0	897.19
VSGILDPI	-1.53	Non-	0.11	1.06	-0.24	0	1054.

KL		AHT					41
LSVAFIAA ML	-1.55	Non-AHT	0.37	2.56	-1.19	0	1035.42

Table S2. parameter of peptides

Peptides	The frequency of bioactive fragments occurrence in protein sequence (A)	Potential biological activity of protein (B)
FAGDDAPR	0.750	0.042
QGPIGPR	0.857	0.037
IFPRNPP	0.714	0.036
AGFAGDDAPR	0.800	0.033
GPTGPAGPR	1.111	0.030
GPAGPAGPR	1.111	0.029452511672
LPPIAF	1.000	0.029130310490
PAAKPLGDL	0.778	0.029024976543
GAQGPIGPR	0.778	0.028886287428
FAPPAPNGVP	0.800	0.028602947680
GSPRAPEGAP	0.700	0.025563516214
AWKPPLQ	0.857	0.021537169640
FDKPVSP	0.429	0.021146462792
FDKPVSPL	0.500	0.018873722949
ILPPGPPTPW	1.100	0.018533232814
FDKPVSPLF	0.556	0.017095012568
NFSLDGPIP	0.444	0.012727750101
FLGLMWFLP	0.444	0.012111400079
GRPGPPGVP	1.111	0.011787078216
NFPPGPPGIP	1.000	0.008066456476
IPGPPTGPIK	0.900	0.007170112579
EAPPHIF	1.000	0.005616546215
IEAPPHIF	1.000	0.004914477938
VILPVPAF	0.625000000000	0.005

RIEAPPHIF	0.889	0.004368424834
KRIEAPPHIF	0.900	0.004194740245
KAEPTPGKM	0.556	0.003939716316
KAEPVPGKM	0.444	0.003818999205
VPIGAFK	0.571	0.001282491944
PGAPGSPGMP	0.700	0.000600174028
AEPVPGKM	0.375	0.000328120137
GVDNPGHPFI	0.400	0.000059879548

Table S3. The 5 peptides were screened

	Allergenicity	Intestinal stability	Half-Life (s)	Molecular weight (Da)	Isoelectric point	Toxicity	Amphiphilicity
FAGDDAPR	NON-ALLERGEN	High	845.510	847.970	5.756	Non-Toxin	0.310
QGPIGPR	NON-ALLERGEN	High	834.410	723.930	6.710	Non-Toxin	0.530
IFPRNPP	ALLERGEN	High	874.210	840.080	6.653	Non-Toxin	0.350
AGFAGDDAPR	ALLERGEN	High	878.810	976.130	5.802	Non-Toxin	0.250
GPTGPAGPR	NON-ALLERGEN	High	775.810	809.010	6.581	Non-Toxin	0.270
